# Developing a Sustainable Quality Improvement Program in an Academic Center: The Experience of an Adolescent Unit

**DOI:** 10.1097/pq9.0000000000000742

**Published:** 2024-06-11

**Authors:** Erin R. McKnight, Ashley Ebersole, James Gallup, Fareeda W. Haamid

**Affiliations:** From the * Division of Adolescent Medicine, Department of Pediatrics, Nationwide Children’s Hospital, Columbus, Ohio; † Department of Pediatrics, The Ohio State University Wexner College of Medicine, Columbus, Ohio; ‡ Center for Clinical Excellence, Nationwide Children’s Hospital, Columbus, Ohio; § Quality Improvement Services, Department of Pediatrics, Nationwide Children’s Hospital, Columbus, Ohio.

## Abstract

**Introduction:**

Successful quality improvement (QI) efforts use a comprehensive, institutional QI framework and professional development, but literature describing implementing QI frameworks in Adolescent Medicine practices is sparse. We aimed to implement and increase the number of formally structured QI projects (primary aim) and the number of projects achieving a centerline (CL) shift (secondary aim) in our hospital’s Adolescent Medicine Clinic.

**Methods:**

We used formal QI methodology to improve health outcomes by increasing the number of faculty with formalized QI education, creating interdisciplinary QI teams, and improving staff motivation. QI education was mandatory for Adolescent Medicine fellows and pediatric residents and encouraged for faculty and staff. The Divisional QI leadership team attended monthly meetings to review key driver diagrams, run and control charts, and receive intervention updates. All providers and staff received monthly updates, and the Hospital Quality and Safety Committee received biannual updates. We used run charts to share progress with primary and secondary aims.

**Results:**

Since Q3 2014, the Adolescent Medicine team consistently achieved the primary aim of having 5 active projects in process, with 9 projects from Q1 2018–Q4 2020. For the secondary aim, a target of 50% of active QI projects attaining a sustained centerline shift was achieved in Q2 2018 and maintained in 16 of 20 quarters since.

**Conclusions:**

Clinicians can use QI methodology to improve health outcomes while facilitating professional development. For this initiative to succeed, institutional leadership must provide an infrastructure prioritizing meaningful QI involvement.

## INTRODUCTION

Although the impact of a nonstandardized approach to systematic quality improvement (QI) is unknown, particularly in an adolescent patient setting, a nonstandardized approach may result in inefficient resource use and provide suboptimal care. Although publications describing singular system-focused QI projects have increased,^1–4^ scant literature describes implementing comprehensive QI programs in large multidisciplinary divisions.^5^

Academic providers often contend with other demands, including administrative roles.^6,7^ Outside patient care, QI may be perceived as another administrative task, receiving lower priority at institutions. Integrating successful QI initiatives targeting health outcomes requires an intentional team effort and meaningful institutional investment.^8–10^ Previous data emphasizing successful QI efforts in various patient populations use a comprehensive institutional QI framework and concerted faculty and staff development.^8,11–16^ We theorized that we could achieve positive adolescent health outcomes following a similar approach.

**Fig. 1.**
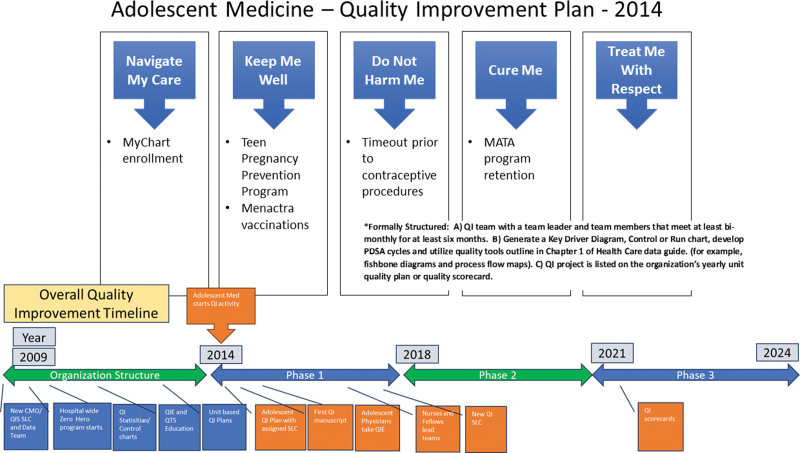
Adolescent Medicine QI Plan. QTS, Quality Tool School; MATA, Medication Assisted Treatment of Addiction.

**Fig. 2.**
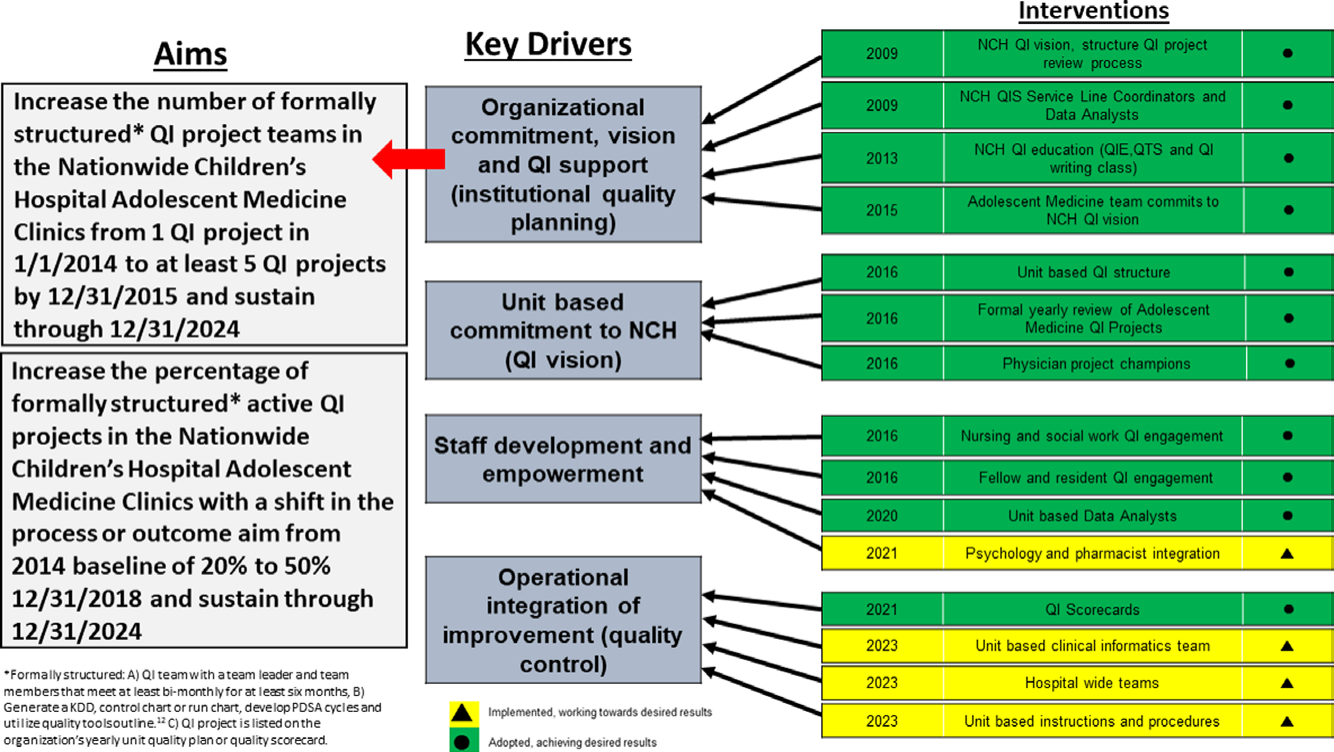
Key driver diagram: Adolescent Medicine QI Project development.

Starting late in 2013, the Nationwide Children’s Hospital (NCH) Adolescent Medicine team committed to developing a unit-based QI plan supported by the hospital’s Quality Improvement Services Department (QIS) and began implementation in 2014. The QIS Team used a structured team QI problem-solving process with methods recommended by the IHI requiring biannual reporting to the Chief Medical Officer (CMO).^17,18^ To describe our QI system development (Fig. 1) and to detail the structured team QI problem-solving process, a retrospective key driver diagram (KDD) was developed to outline our 10-year journey to date (Fig. 2).

## METHODS

### Context and Setting

We conducted this project within the Adolescent Medicine Clinic at NCH, a nonprofit, freestanding academic hospital. The Adolescent Medicine Clinic provides primary care, specialized reproductive healthcare, eating disorder treatment, and substance use services for patients aged 11–25. Our clinic volume averages 11,000 annual visits. The clinic team includes Adolescent Medicine attending physicians and fellows, nurses, medical assistants, advanced practice nurse practitioners, registration staff, social workers, clinical psychologists, a pharmacist, and rotating trainees.

The hospital’s strategic plan focuses on *Leading the Journey to Best Outcomes*, where Quality, and Safety are key tenets.^9,19,20^ QI efforts at NCH are a robust part of organizational culture, operationalized in day-to-day institutional activity. From senior management’s QI leadership to the data infrastructure, resource availability, and workforce focus on QI, organizational support is bountiful, and interdisciplinary collaboration is expected.^13,15,16^

### Ethics

The institutional review board deemed this project QI and not human subjects’ research; thus, it did not require their review and approval.

### Interventions

#### Organizational Commitment, Vision, and QI Support (Institutional QI Framework)

In 2009, NCH QIS leadership and NCH Executive leadership committed to an enhanced vision of team-based QI based on IHI methodology.^3,21–23^ (Fig. 1) The Zero Hero initiative provided a new platform for developing an enhanced QI culture that involved all levels of the organization and established enhanced unit-based collaborative QI teams.^20,24,25^ QI support was provided by establishing QI Service Line Coordinators (SLC), from varied backgrounds.^26^ The SLCs were assigned to hospital units or collaborative teams to teach QI skills and facilitate teams with a long-term goal of empowering teams to participate in and own various QI projects.^7,8,27,28^ To support data needs for improvement efforts, the QIS team integrated data analysts responsible for developing QI team reports and a QI statistician to implement a statistical process control charting strategy.^29^ In 2011, two formal QI educational programs were developed to support the expanding number of QI teams. The 40-hour project-based Quality Improvement Essential (QIE) Course targeted the physician and nursing leaders committed to expanding QI initiatives and formalizing a QI structure.^8,30^ Additionally, a condensed educational program, the Quality Tool School, was established for all employees unable to participate in the QIE class, which supported QI skills and activities.^2,13^

#### Unit-based commitment and vision

**Fig. 3.**
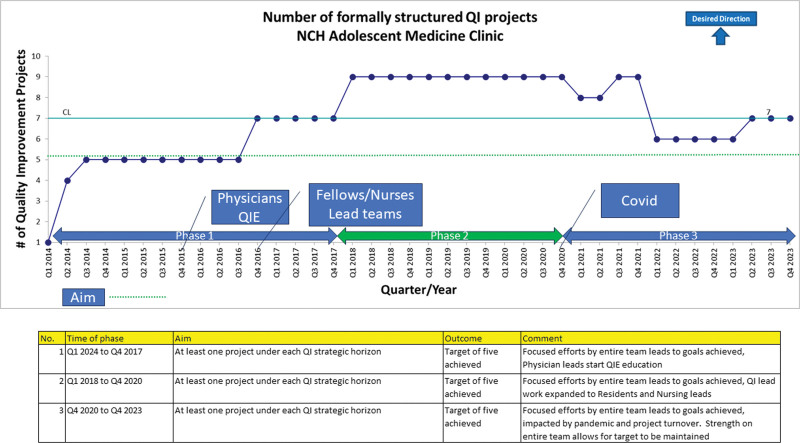
Number of formally structured QI projects in Adolescent Medicine Clinic.

**Fig. 4.**
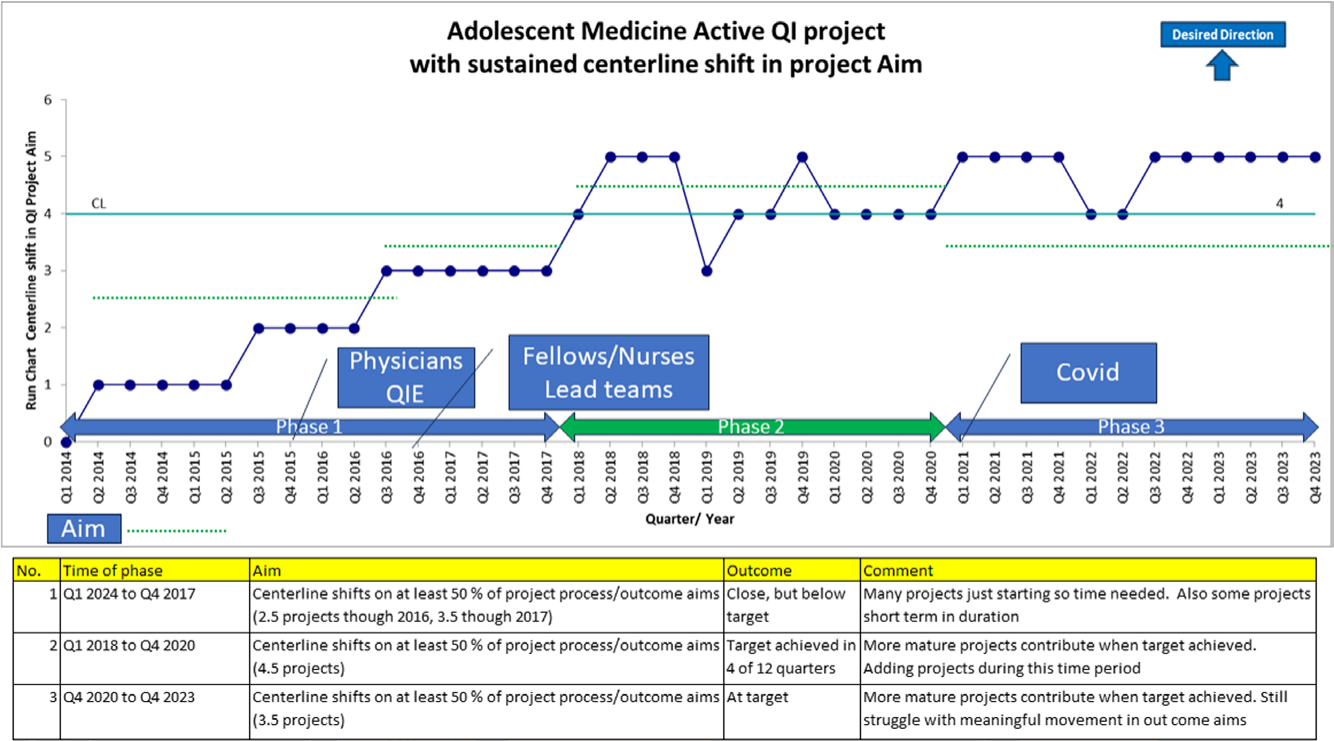
Percentage of Active Adolescent Medicine QI projects with a sustained centerline shift.

The Adolescent Medicine team committed to unit-based quality activities and was assigned their first QIS SLC in late 2013. This vision included developing projects under 5 strategic horizons: (1) Navigate My Care, (2) Keep Me Well, (3) Don’t Harm Me, (4) Cure Me, and (5) Treat Me With Respect (Fig. 1). They also established a unit-based QI lead physician who completed the QIE course to develop and lead at least one QI project. Finally, a unit-based quality structure was created within the division to foster QI education, review QI projects, and develop new multidisciplinary teams and projects. Progress reports were presented biannually to the CMO and QIS leadership, which impacted the growth of a QI culture in many ways, including a review of ongoing project KDD and control chart data and how projects related to the five strategic horizons. For retrospective QI system development discussion, we developed a KDD (Fig. 2), two run charts (Figs. 3 and 4), and a detailed project table (**Supplemental Table,** http://links.lww.com/PQ9/A564).

#### Staff Development and Empowerment

In 2016, along with existing QI projects, the Adolescent Physician team committed to adding 4 interventions to the QI portfolio. (Figs. 3 and 4) This endeavor was possible due to integrating more physician project leaders and the inclusion of fellows, residents, nursing, and social work staff into regular QI team meetings and interventions. All physician champions and staff used formal QI education throughout the development of their QI projects. In 2017, based on feedback obtained in biannual reviews, the adolescent team incorporated the following strategies: focusing on 6 QI projects to impact clinical improvement and placing other QI projects that had met goals into sustain mode. In 2018 and 2019, the Adolescent QI team established 3 initiatives that broadened the scope outside the division. The THRIVE and Nurse Led Depo initiatives targeted cross-functional work involving partners outside the Adolescent Division. In addition, with the support of an Adolescent faculty physician, the Transition of Care project became the first fellow-led project. The Transition of Care project expanded outside the division and included hospital-wide improvement efforts. Information from various projects was disseminated through publications, NCH Pediatric Grand Rounds, and the nursing team shared project results at the Chief Nursing Officer’s (CNO) annual Nursing Congress meeting.^31–33^

#### Operational Integration of Improvement

**Fig. 5.**
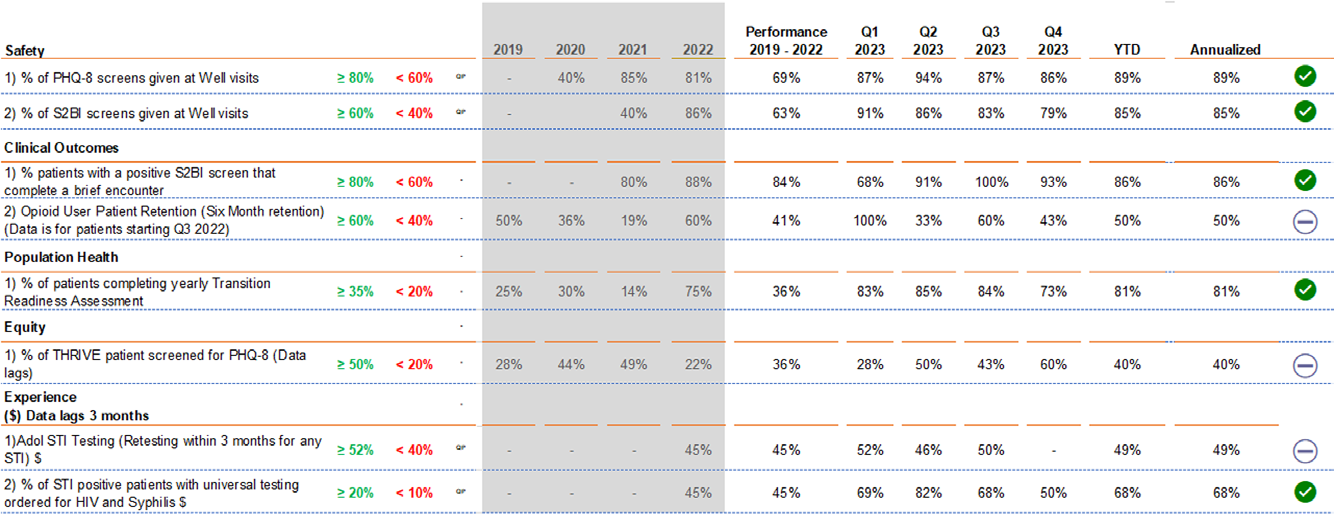
Quality Scorecard: Adolescent Medicine QI Project development. PHQ, Patient Health Questionnaire; S2BI, Screening to Brief Intervention; YTD, year to date.

In 2021, the Center for Clinical Excellence (CCE) was established with new medical leadership, which resulted in an innovative direction for QI. The yearly reports to the CMO and CCE leadership now included specific Quality Plan/Quality initiatives with a quality scorecard to reflect the CCE strategic initiatives. (Fig. 5) The change resulted in a more selective approach to retiring and starting projects and more focus on meeting yearly targets set by the QI leadership team.

##### Future Directions.

 The Adolescent Medicine Division highlighted the mental health crisis, including adolescent substance use, which necessitated additional support in the form of improved data analysis and clinical informatics education for physician leaders. This expanded education was integrated within a larger hospital-wide QI initiative to increase behavioral health screening and treatment, a major strategic initiative for NCH that involved a wide array of division and multidisciplinary team members. The unit-based informatics will enhance the development of standard work necessary for ongoing improvement in quality control.

### Analysis

A QI project required an employee champion, a QI team, a control chart with aim data, a KDD, and targeted interventions to improve the aim. We monitored the progress of KDD-based QI projects during monthly Adolescent Medicine QI Leadership review meetings. In addition, we routinely shared meeting feedback with QI teams to assist teams with accomplishing focused aims.

The team studied process changes over time based on data loaded into proprietary SPC charting templates.^34^ We applied the American Society for Quality criteria to adjust the CL and evaluated each of our outcomes quarterly.^29^ We modified interventions monthly as needed based on PDSA cycles and feedback. The team used run charts with results shared below to evaluate the retrospective Key Driver.

## RESULTS

### Primary aim

The primary aim was to implement and increase the number of formally structured QI projects in the NCH Adolescent Medicine Clinics, which began in Q1 2014 (Fig. 3). The QI physician lead completed the QIE course in Q4 2015, followed by 3 additional Adolescent Medicine physicians from 2017 to 2018. We used a run chart (Fig. 3) to track this aim. The team consistently supported 5 projects during the initial portion of phase 1 (Q1 2014 to Q4 2017). Due to increased team and physician activity, projects increased to seven starting Q4 2016 and nine projects throughout phase 2 (Q1 2018 to Q4 2020). The COVID pandemic impacted projects during phase 3 (Q1 2021 to Q4 2023); however, the team rebounded to seven projects starting in Q2 2023, aligning with the new QI scorecard strategy.

A supplemental table with all of the Division’s QI projects that achieved a sustained centerline shift and their impact is included for further reference.

### Secondary Aim

A secondary aim was to increase the percentage of active QI projects achieving a sustained CL shift in a process or outcome aim. A run chart (Fig. 4) was used to track this aim. The team showed steady progress on this aim during phase 1 when many of these projects were starting. The breakthrough in this aim occurred in Phase 2 due to increased team involvement and project maturity. The team achieved the 50% target in four of 12 quarters in phase 2 and maintained a centerline average of 4 projects with a centerline shift. As our QI team projects matured and even through the COVID-19 pandemic, Phase 3 saw the team consistently at goal on the secondary aim with five projects with sustained centerline shifts.

## DISCUSSION

Emphasis on a formalized approach to QI education and initiatives created an important culture change within the Division of Adolescent Medicine that improved the way physicians, nurses, social workers, trainees, and staff worked together and approached problem-solving. This culture change focused on QI led to improved adolescent health outcomes to generate and execute formally structured QI projects. Continuing to perpetuate a QI culture within a multidisciplinary division facilitated by team members’ interests, roles, capacity to participate, and ongoing feedback allowed us to expand our goals and increase the number of QI projects with sustainable CL shifts over 8 years.

### Factors That Led to Success

Key factors that led to our success were sufficient institutional support, formalized QI team education, and cycles of relevant new projects. Our divisional QI model encouraged physicians, nurses, and ancillary staff to engage in formal QI education, improving the knowledge gained from one QI project and applying it to future multidisciplinary projects. Formal QI education for an interdisciplinary team was the cornerstone of building a culture of sustained engagement in QI efforts.^12^

Nonphysician team member’s participation was critical to our QI culture and driving change. We needed the unique expertise of all team members specific to their training to generate and sustain new impactful QI projects that pertained to the entire patient experience. Nurses commonly sought QI opportunities to enhance their professional development and career advancement, including the Quality Tool School and readily engaged in projects that garnered their enthusiasm. Their input was integral to implementing projects addressing contraceptive access, mental health surveillance, and preventative care visits. Nurses were solely responsible for executing various QI projects, which enabled them to practice at the uppermost scope of their professional licenses. For example, nurses participated in our nurse-led contraceptive visit project and reported this role expansion improved job satisfaction. Their input was critical to combat the day-to-day barriers experienced by nurses and patients during routine contraception visits.

The social work team was also a beneficiary of formalized QI training, which led to their involvement in multiple QI projects. Their integral engagement in the substance use treatment program’s various QI efforts led to increased patient retention and overall sustained recovery of patients with opioid use disorder.

### Factors That Inhibited Success

Our effectiveness greatly diminished when we did not account for the flexibility needed in the context of competing academic demands when a QI project was added more for quantity as opposed to quality and at times when we overly relied on a few team members. Thus, we highly recommend that teams and their leaders anticipate the inherent dynamic nature of QI processes on the teams who execute these projects.

In addition, while integrating multidisciplinary staff led to our division’s QI achievements, staff turnover was a significant barrier to success. While continuing to onboard staff members to the unique clinical attributes of adolescent health care, our team also had to emphasize the importance of QI education and its impact on adolescent health. To improve the QI work within a clinical division, it is vital to formally educate new team members and ensure a continued emphasis on QI within the divisional culture.

Finally, a working knowledge of when to place a project in sustain mode is critical to minimize team burnout, maximize our efforts and expand our capabilities. A project that reaches its stated goals can be retired, allowing for a new project championed by an enthusiastic team member to be introduced. Our division was initially slow to place projects in sustain mode, creating barriers around time for team members to formulate new ideas and projects. Once our team learned the importance of when to put projects in sustain mode, it opened up the opportunity for team members to create new projects. This necessary cycle of fresh ideas bolsters team confidence and focuses on the most pressing adolescent health challenges.

#### Ensuring Physician Engagement

Establishing a physician lead, physician-generated projects, Division Chief support, and QIE course involvement were dynamic processes that facilitated physician buy-in. The Division Chief worked closely with the physician lead to identify potential QI projects. The Chief also monitored physician engagement to ensure it was aligned with the institutional expectations and strategic plan. The Chief strove to improve patient health and mitigate physician burnout by progressively emphasizing high-quality projects rather than project quantity.

The physician leader strategically prioritized QI in the division and was provided with ongoing institutional resources. This physician was vital to the comprehensive QI program by directing and supporting multiple projects to ensure the divisional QI portfolio was adequately balanced.

Physicians who completed the QIE course became proficient team leaders, allowing divisional QI responsibilities to be evenly distributed. Institutional flexibility was necessary during increased physician demands, such as staffing changes and the high stress at the onset of the COVID-19 pandemic in 2020. Ultimately, we enhanced physician involvement by turning QI projects into journal publications and presentations at professional meetings. This coupling allowed for academic productivity, sustaining physician engagement.^31,32,35^

#### Effective Team Management

Behavioral economics is an underutilized tool for maximizing QI participation in health care.^36^ Although initially unintentional, our team found engagement of the entire interdisciplinary team and a strengthened culture of QI participation by employing behavioral economics principles. It, therefore, continued to use strategies such as salience and descriptive norms.

#### Use of Extensive QI Institutional Framework

The QI climate change in the Adolescent Medicine Division had far-reaching effects. Team members joined more complex hospital QI initiatives, including transitioning to adult health care, opioid stewardship, and decreasing infant mortality. These collaborative efforts helped maintain adolescent health issues at the forefront of hospital and local policies, promoted networking, and enabled Adolescent Medicine team members to interact with the community and institutional stakeholders. New partnerships also led to funded grant proposals, allocation of foundation donor funds, and ongoing scholarly pursuits. Plans include integrating divisional QI efforts into larger hospital QI goals via a standardized scorecard and an advanced overview of long-term strategic goals and outcomes.^37^

### Clinical Impact

The formalization of QI in the Adolescent Medicine Division lead to projects to improve patient safety, experience, clinical outcomes, and population health. Projects focused on safety included increasing the number of Patient Health Questionnaire-9 depression screens given at well visits, which achieved a benchmark directional shift. Improved clinical outcomes were seen with formalized initiatives in the Substance Use Treatment and Recovery program to improve patient retention, with initially improved clinic second visit return and 6-month retention rates, and later increased one and two-year retention.^31^ This is important as previous research highlights that while retention within the program was initially difficult, it was the single factor associated with medication for opioid use disorder compliance and opioid abstinence.^33^ Patient experience has improved, with more patients performing a transition readiness assessment and starting a formalized process of moving toward adult care. In addition, structured projects related to nurse-led depo-medroxyprogesterone visits allow patients to receive this effective and safe contraception within a shorter visit, allowing them to return to their daily activities sooner. Adolescent population health is also a priority for QI projects, including formalized efforts to improve sexually transmitted infection screening of adolescents 3 months after testing positive for a sexually transmitted infection.

### Limitations

Institutions with less robust QI infrastructure may find implementing QI efforts challenging, and team member engagement may fluctuate. Similarly, other divisions may not have the capability for interdisciplinary QI teams. In addition, staff turnover may hinder the progress of projects. For example, we experienced palpable setbacks at times of nursing staff turnover. Additionally, data abstraction capabilities from the electronic medical record, which strengthened this work, may not be available at all institutions.

## CONCLUSIONS

This work is the first to detail a process framework for a comprehensive approach directed at multiple adolescent-specific QI projects involving an expansive team and stakeholders in the context of adequate institutional support. Although this framework is in an ideal environment, the authors acknowledge the complexities of implementing QI in an academic setting and the dynamic nature of healthcare utilization patterns. Nonetheless, the description of the QI implementation process and the subsequent outcomes of comprehensive QI are worthwhile and paramount to advancing optimal healthcare delivery.

Importantly, time and effort should be placed on creating a culture within a division where QI is encouraged, and multidisciplinary team education and involvement are a mainstay of approach to problem solving and improving health outcomes. This vital culture change will allow for improved teamwork and generate new ideas that enable formalized QI projects to thrive and ultimately improve health outcomes.

Clinicians should adapt interventions based on their institutional capabilities, and institutional leadership must build supportive frameworks prioritizing meaningful QI involvement. Implementing a comprehensive QI program targeting multiple health outcomes is dynamic and challenging, but opportunities for advancements in patient care and professional development are immense.

## ACKNOWLEDGMENTS

The authors thank the faculty and staff in the Division of Adolescent Medicine at NCH for their ideas, effort, and support of QI. The authors also thank our former QIS service line coordinator Stephanie Lemle and the NCH QI Writing Group, notably Drs. Melody Davis, Sandra Spencer, Robert Gajarksi, Christina Toth, and Veronica Mruk for their review of article drafts.

## Supplementary Material



## References

[1] Forman-Hoffman VL Middleton JC McKeeman JL. Quality improvement, implementation, and dissemination strategies to improve mental health care for children and adolescents: a systematic review. Implement Sci. 2017;12:93. 28738821 10.1186/s13012-017-0626-4 PMC5525230

[2] Gallup J Buckingham D Dolan K. Quality tool school: improving the delivery of quality improvement education in a children’s hospital. Pediatr Qual Saf. 2023;8:e680. 37780601 10.1097/pq9.0000000000000680 PMC10538879

[3] Huguelet PS Olson E Sass A. Application of a standard cross-specialty workup for diagnosis and metabolic screening of obese adolescents with polycystic ovary syndrome. J Adolesc Health. 2021;68:589–595. 32819830 10.1016/j.jadohealth.2020.07.008

[4] DiVasta AD Trudell EK Francis M. Practice-based quality improvement collaborative to increase chlamydia screening in young women. Pediatrics. 2016;137:e20151082. 27244777 10.1542/peds.2015-1082

[5] Riley M Patterson V Lane JC. The adolescent champion model: primary care becomes adolescent-centered via targeted quality improvement. J Pediatr. 2018;193:229–236.e1. 29198766 10.1016/j.jpeds.2017.09.084

[6] Shah DT Williams VN Thorndyke LE. Restoring faculty vitality in academic medicine when burnout threatens. Acad Med. 2018;93:979–984. 29166355 10.1097/ACM.0000000000002013 PMC6169302

[7] Lieff SJ Yammarino FJ. How to lead the way through complexity, constraint, and uncertainty in academic health science centers. Acad Med. 2017;92:614–621. 28441672 10.1097/ACM.0000000000001475

[8] Bartman T Heiser K Bethune A. Interprofessional QI training enhances competency and QI productivity among graduates: findings from nationwide children’s hospital. Acad Med. 2018;93:292–298. 28817428 10.1097/ACM.0000000000001862

[9] Levy FH Brilli RJ First LR. A new framework for quality partnerships in Children’s Hospitals. Pediatrics. 2011;127:1147–1156. 21576310 10.1542/peds.2010-1409

[10] Garman A Scribner L. Leading for quality in healthcare: development and validation of a competency model. J Healthc Manag. 2011;56:373 82; discussion 382. 22201200

[11] Aronson MD Neeman N Carbo A. A model for quality improvement programs in academic departments of medicine. Am J Med. 2008;121:922–929. 18823865 10.1016/j.amjmed.2008.06.018

[12] Bartman T Brilli RJ. Quality improvement studies in pediatric critical care medicine. Pediatr Crit Care Med. 2021;22:662–668. 34192729 10.1097/PCC.0000000000002744

[13] Bode RS Brilli RJ. Health care quality improvement fluency: the secret sauce to improving quality improvement. Ann Allergy Asthma Immunol. 2023;130:550–551. 36519724 10.1016/j.anai.2022.11.023

[14] Kraft S Carayon P Weiss J. A simple framework for complex system improvement. Am J Med Qual. 2015;30:223–231. 24723664 10.1177/1062860614530184 PMC4193943

[15] Ralston SL Brady PW Kemper AR. Do we really need scholarly quality improvement? JAMA Pediatr. 2019;173:413–414. 30830176 10.1001/jamapediatrics.2019.0067

[16] Ralston SL Holmes AV Gautham KS. Do we really need a scholarly quality improvement workforce? Pediatrics. 2022;149(Suppl 3):e2020045948F. 10.1542/peds.2020-045948F 35230432

[17] Provost LP Murray SK Holts A. The health care data guide: learning from data for improvement. 1st ed. New York Academy of Sciences Ser. Jossey-Bass Wairīpaburisshingujapan; 2011.

[18] Langley G Moen R Nolan K. *The Improvement Guide: A Practical Approach to Enhancing Organizational Performance.* Second ed. Jossey-Bass; 2009.

[19] Hospital NCs. Fast facts. Available at https://www.nationwidechildrens.org/about-us/our-story/fast-facts. Accessed July 25, 2023.

[20] Brilli RJ McClead RE Jr Crandall WV. A comprehensive patient safety program can significantly reduce preventable harm, associated costs, and hospital mortality. *J Pediatr.* 2013;163:1638–1645. 23910978 10.1016/j.jpeds.2013.06.031

[21] Institute for Healthcare Improvement. IHI Open School. Available at https://www.ihi.org/education/ihi-open-school. Accessed January 29, 2024.

[22] Juran on Leadership for Quality: an Executive Handbook. New York: Free Press Collier Macmillan; 1989. Available at: http://catdir.loc.gov/catdir/enhancements/fy0705/88021306-t.html. Available at: http://catdir.loc.gov/catdir/samples/simon031/88021306.html.

[23] Kaminski GM Schoettker PJ Alessandrini EA. A comprehensive model to build improvement capability in a pediatric academic medical center. Acad Pediatr. 2014;14:29–39. 24369867 10.1016/j.acap.2013.02.007

[24] McClead RE Jr Catt C Davis JT; Adverse Drug Event Quality Collaborative. An internal quality improvement collaborative significantly reduces hospital-wide medication error related adverse drug events. J Pediatr. 2014;165:1222–1229.e1. 25304926 10.1016/j.jpeds.2014.08.063

[25] The Center for Clinical Excellence. Hospital NCs. Available at https://www.nationwidechildrens.org/impact-quality. Updated January 29, 2024.

[26] Bens I. Facilitation at a glance!: a Pocket Guide of Tools and Techniques for Effective Meeting Facilitation. 2nd edition ed. Goal/QPC; 2008;vi:181 pages: illustrations.

[27] Dennis P. Andy & Me and the Hospital: Further Adventures on the Lean Journey. New York: Productivity Press; 2016:1–247.

[28] Hersey P. The Situational Leader. Center for Leadership Studies; 1984:126 pages: illustrations.

[29] Carey RG. Improving Health Care with Control Charts, Basic and Advanced SPC Methods and Case Studies, American Society of Quality Control. ASQ Quality Press; 2003.

[30] Dean MLM Rich L. The Lean Memory Jogger for Healthcare. Goal/QPC; 1999.

[31] Cottrill CB Lemle S Matson SC. Multifaceted quality improvement initiative improves retention in treatment for youth with opioid use disorder. Pediatr Qual Saf. 2019;4:e174. 31579873 10.1097/pq9.0000000000000174 PMC6594786

[32] Ebersole AM Gallup J Rockwell A. Implementing evidence-based, electronic, substance-use screening in a primary care clinic. J Adolesc Health. 2023;73:127–132. 37031088 10.1016/j.jadohealth.2023.02.010

[33] Matson SC Hobson G Abdel-Rasoul M. A retrospective study of retention of opioid-dependent adolescents and young adults in an outpatient buprenorphine/naloxone clinic. J Addict Med. 2014;8:176–182. 24695018 10.1097/ADM.0000000000000035

[34] QI Macros. KnowWare International I. Available at https://www.qimacros.com/

[35] Balch Samora J Spencer SP Valleru J. Writing group increases quality improvement writing competency. Am J Med Qual. 2020;35:349–354. 31718231 10.1177/1062860619886910

[36] Stevens J. The promising contributions of behavioral economics to quality improvement in health care. Pediatr Qual Saf. 2017;2:e023. 30229161 10.1097/pq9.0000000000000023 PMC6132457

[37] Lowder D. Healthcare dashboards vs. scorecards: use both to improve outcomes. Health Catalyst. Available at https://www.healthcatalyst.com/healthcare-dashboards-vs-scorecards-to-improve-outcomes. Updated May 22, 2018.

