# Supplementary material for: Developing a Sustainable Quality Improvement Program in an Academic Center: The Experience of an Adolescent Unit
Source: Pediatr Qual Saf. 2024 Jun 11;9(3):e742. doi: 10.1097/pq9.0000000000000742 (PMC11167228; doi:10.1097/pq9.0000000000000742)
Supplement: Supplementary file 1 [file pqs-9-e742-s001.pdf]

No.	Project	Quarter (Q) started	Quarter Completed	Project Aims (Outcome and Process)		Aim achieved	Statistical shift	Impact	Publication date
1	MyChart	Q1 2014	Q4 2018	Among patients seen in Adolescent Medicine clinic who identify Adolescent Medicine as their primary care provider, increase “active” status in MyChart from 6.7% in 2013 to 40% by end of Q4 2016 and maintain for 6 months		Yes	Yes	Spread the use of the "My Chart" EPIC based tool enhancing communication between patients and clinical staff.	
2	Teen pregnancy prevention	Q1 2014	Q4 2015	1) Reduce the birth rate in 15-19 year old females in the Healthy Neighborhoods, Healthy Families zone by 20% from 58.46 per 1000 in 2012 to 46.77 per 1000 in 2017	2) Increase the number of patients receiving a long-acting reversible contraceptive method in Adolescent Medicine clinic by 25% compound annual growth rate from 229 in 2013 to 559 in 2017	1) No<br>2) Yes	1) No<br>2) No	Beginning projects to address hospital wide teen pregnancy challenges. Project was springboard for hospital wide efforts.	
3	Immunization, well Care improvement	Q1 2014	Q4 2018	1) Increase number of well care visits in Adolescent Medicine clinic, for patients ages from 334 in 2015 to 355 in 2016 by Aug 31, 2016 and sustain for 1 year	2) Increase administered meningococcal, HPV and Tdap vaccinations for patients seen in Adolescent Medicine	1) Yes<br>2) Yes	1) No<br>2) TDAP only	Increased awareness of hospital wide targets for immunization and well care visits.	

					clinic who have identified an Adolescent Medicine provider as their PCP or “no PCP” (□16 years of age for meningococcal, □14 years of age for Tdap & □9 years of age for HPV) by 5% by end of Q2 2017 and maintain for 6 months.				
4	Substance use treatment program retention	Q2 2015	ongoing	1) Improve the 6-month long-term retention rate from 19% in 2012-2013 to 35% by end of Q4 2017 for patients in the substance use treatment program	2) Improve the one year and two year long-term retention rate from 19% in 2012-2013 to 35% by end of Q4 2017 for patients in the substance use treatment program	1) Yes<br>2) Yes	1) No<br>2) One year rate only	Increased retention of patients in clinic, data from publication helped secure $500,000 in donor funds from institution foundation.	2014, 2019
5	Timeouts prior to contraceptive procedures	Q3 2014	Q3 2016	For contraception procedures done in Adolescent Medicine clinic, increase completion and documentation of pre-procedure time outs from 90% in 2013 to 100% by end of Q4 2016 and maintain for 6 months		No	No	Improve safety and process around time outs, first Zero Hero based project for team.	

6	Sexually transmitted infections (STIs) recurrence	Q1 2015	Q4 2020	Decrease the recurrence of STIs (Chlamydia, gonorrhea, and Trichomonas) in all adolescent patients presenting to Adolescent Medicine clinic who have had an STI within the past 6 months from a baseline of 23% to 13% by end of Q4 2016 and sustain for 6 months	No	No	QIE project for lead physician, presented challenges to achieve targets based difficulties with target population. Multiple PDSA's tried, some success, however no longer term metric progress.	
7	Eating disorders	Q4 2016	Q4 2021	Increase compliance of assessment bundle within Eating Disorders Program for diagnostic intake, from 10% to 50% and then to 90%, by June 2020 and sustain for 12 months	Yes	Yes	Very successful efforts to improve overall treatment for eating disorders patients.	
8	Estrogen contraindications	Q4 2016	Q4 2018	Increase standardized documentation of estrogen contraindications in Adolescent Medicine clinic visits where an estrogen containing medication is prescribed or continued from 20% to 100% by 7/31/17 and sustain for 6 months	No	No	Standardized documentation now in all note templates and included in all visits where estrogen is prescribed.	
9	BC4Teens (Birth control for teens)	Q4 2016	Q4 2019	Increase show rate in BC4Teens clinic from 46% to 60%, by end of Q2 2017 and sustain for 6 months	Yes	Yes	Very effective interventions implemented by nursing team and physicians to increase clinic flow challenges.	

10	Transgender clinic (THRIVE)	Q1 2018	ongoing	1) Decrease average total days to first, second and third appointment for simple and complex THRIVE patients by 10% by Q4 2019 and reduce 20% by Q4 2020	2) Increase the number of THRIVE patients screened for depression (PHQ-8) from baseline of 36% to 50% by December 2022 and sustain for 1 year	1) Yes<br>2) No	1) Yes<br>2) No	First cross departmental project with Adolescent Medicine and Psychology. Increased awareness of overall appointment flow for the transgender population.	
11	Nurse led depo medroxyprog esterone acetate visits	Q1 2018	Q4 2020	Increase the percent of visits during which depo medroxyprogesterone acetate is administered that are “nurse-led visits” among patients seen in Adolescent Medicine clinic from 0% to 25% by November 1, 2018 and sustain for one year		Yes	No	QIE project for lead physician, very effective interventions implemented by nursing team and physicians.	
12	Depression screening	Q1 2019	ongoing	Increase completed PHQ-9 screening forms for patients 12 to 22 years of age for targeted visits from 0% to 20%, by end of Q1 2020 and to 50% by end of Q4 2022		Yes	Yes	Project underscores the importance of integrating electronic based patient forms.	
13	Transition of care	Q1 2019	ongoing	Increase the number of patients who present for a well visit for all providers who receive a modified five question transition of care questionnaire, from 0% to 60% by 1/2021 and sustain for six months		Yes	Yes	Part of a hospital wide effort to standardize use of the Got Transition® screener for transition of care. Team expanded use of iPad screening to	

								improve overall use of the screener.	
14	PDSA nursing projects	Q1 2019	Q1 2022	Increase the number of nurse based short cycle interventions for Adolescent Medicine clinic from 1 per quarter to 2 per quarter by end of Q4 2019 and sustain through 2020		Yes	No	First nursing led project. Intent was involve nurses more directly in structured PDSA short cycles to apply what was learned as they completed Quality Tool School. Shared learning with Chief Nursing Officer and nursing staff.	
15	Telehealth	Q1 2020	Q1 2022	Increase the percentage of adolescent visits that are telehealth in Adolescent Clinics from a 2020 baseline of 0% to a monthly average of 10% by end of Q4 2020 and sustain through Q2 2021		Yes	No	Target interventions during the COVID-19 pandemic, part of hospital wide efforts to expand telehealth visits.	
16	STI screening	Q1 2021	ongoing	1) Increase the percentage of patients with positive STIs who are rescreened within 16 weeks of initial positive screening in PAG and Adolescent Medicine clinic from 54% to	2) Increase the percentage of positive STI patients with universal testing (HIV and Syphilis) in Adolescent Medicine clinic from 15% to 20%,	1) Yes<br>2) Yes	1) Yes<br>2) No	Increased adolescent physician rescreening of patients who were positive for STIs and comprehensive testing per CDC recommendations for those who were	

				64%, by end of Q2 2023 and sustain through end of Q4 2024	by end of Q4 2023 and sustain through Q4 2024			positive for STI. Increased education to trainees on adolescent rotation regarding screening recommendations.	
17	S2BI screening	Q3 2021	ongoing	1) Increase the number of patients 12 - 17 years of age who complete the S2BI/CRAFFT for targeted visits from 0% to 50%, by end of Q2 2022	2) Increase the number of patients with a positive S2BI/CRAFFT score that complete a brief counseling encounter from 60% to 80%, by end of Q4 2023 and sustain for one year	1) Yes<br>2) Yes	1) Yes<br>2) No	Increased identification and linkage with treatment for patients with substance use. Screening spreading to other NCH clinics.	2023
